# Authors’ Reply to: VanderWeele *et al.*, Chiolero, and Schooling *et al.*

**DOI:** 10.1093/ije/dyw163

**Published:** 2016-08-14

**Authors:** Alex Broadbent, Jan Vandenbroucke, Neil Pearce

**Affiliations:** 1 Department of Philosophy, University of Johannesburg; 2 Leiden University Medical Centre, Department of Clinical Epidemiology, AND Department of Clinical Epidemiology, Aarhus University Hospital; 3 Department of Medical Statistics, London School of Hygiene and Tropical Medicine

We thank Vanderweele and co-authors for their letter.^1^ We wish to discuss three points in response.

First, Vanderweele *et al.* contend that we attack a straw man by lumbering the restricted potential outcomes approach (RPOA) with a commitment to defining causal effects in relation to humanly feasible interventions, when in fact the requirement is only that interventions be well defined, and not that they be humanly feasible.

We respond:

- that in our paper we explicitly pointed out that a commitment to humanly feasible interventions is not logically implied by the RPOA;
- that nevertheless, much of the theoretical work and pedagogy in this field restricts itself in practice to humanly feasible interventions in its choice of examples; and
- that the root cause of much confusion is that the notion of an intervention is entirely unclear in the RPOA.

The last point (iii) bears emphasis. What is an intervention? What makes an intervention well defined?

Among epidemiologists it is common to reserve the term ‘intervention’ for humanly feasible policy or medical actions. It is clear from their letter that Vanderweele *et al.* do not mean this. But then it is not clear what exactly is meant by ‘intervention’ or ‘well defined.’

In the RPOA literature, the notion of a well-defined intervention is central. According to the RPOA, the causal effect of an exposure is not well defined unless an intervention on the exposure of interest is well defined.^2^ In RPOA writings, it is seen as highly problematic to seek to estimate the effect of an exposure by simply specifying the hypothetical alteration of an exposure, without specifying an intervention upon that exposure. We are told that one cannot simply estimate the effect of being obese (rather than normal weight) on mortality;^2^; one must specify the obesity-reducing intervention one has in mind, since different interventions may differently affect mortality.

Similarly, we are told that one cannot directly estimate the effect of being one race rather than another, because there are ‘no reasonable hypothetical interventions when race itself is the exposure’.^3^ (We follow this literature in using the term ‘race’ and set aside controversies about race and ethnicity,^4^ If ‘race’ is contested, ‘ethnicity’ could be inserted instead.) One can get around this problem either by substituting something like ‘the race perceived on a job application, which can be hypothetically manipulated’;^3^ but for a ‘general interpretation of race’,^3^ one must estimate it as the residual effect after the effects of various ‘more manipulable factors’ are subtracted.^3^ The same strategy is also recommended for ‘other non-manipulable exposure [sic] such as sex’.^3^ Thus we conclude from RPOA writings that for a direct estimate of a causal effect of an exposure variable on an outcome variable, one must specify an intervention on the exposure—and define it well.^2^.

Given this reliance on the notion of a well-defined intervention, the notion of intervention itself has not been adequately defined. In particular, we have nowhere else seen such a clear statement as in this letter that human feasibility is not part of what is meant by ‘manipulable’, ‘reasonable hypothetical intervention’ and similar expressions. We welcome the clarification.

Even with this clarification, the notion of ‘well-defined intervention’ is still inadequately defined. ‘One hour of physical exercise a day’ is held up as a well-defined intervention on obesity with respect to mortality.^2^ But it appears to us to be no better specified (and perhaps worse) than the contrast between being obese [e.g. as indicated by a body mass index (BMI) of 35] or non-obese (e.g. BMI of 25), given the various physiological effects of different kinds of exercise. Likewise we are not convinced that contemplated interventions on socioeconomic status^3^ are really any more uniform or less varied in the diversity of their potential outcomes (nor, for that matter, more humanly feasible) than interventions on race itself.

Unless ‘intervention’ is defined, introducing that notion does not help solve the problem of guaranteeing uniform potential outcomes of adjustments to exposure variables. Unless something more is said about them, interventions are just surgical adjustments of certain other variables (exercise habits, socioeconomic status); so why not surgically adjust the exposure variable (obesity, race) itself? A uniform potential outcome is guaranteed in neither case.

Moreover, until ‘intervention’ is defined, there is a lack of clarity in the RPOA itself: in what exactly is being asserted, and on exactly what basis some effect estimates are disallowed—while others are permitted—the title ‘causal’. We worry in particular that the approach will be understood as limiting epidemiological research to the investigation of humanly manipulable factors, because of the common use of ‘intervention’ to mean something one can actually do, because of the use of language that permits or suggests this interpretation in various RPOA writings and because of the lack of worked examples that do not fit this paradigm.

Second, Vanderweele *et al.* object to our introduction of a new term (RPOA). To our knowledge, no equivalent view to theirs is expressed elsewhere in the literature. Their view differs from well-known interventionist views such as those of Judea Pearl or James Woodward, since those views allow any logically possible adjustment of a value of a variable as an intervention^5^ or a surgical incision in a directed acyclic graph (DAG).^6^ Thus they would see no intrinsic difficulty in defining and computing effects of obesity status or race, although they would doubtless insist that the hypothetical intervention be done to a causal model that properly represented the various relationships between the variables. Proponents of the RPOA have, in contrast, argued that it is difficult or impossible to estimate causal effects for obesity status or race because of the absence of well-defined interventions upon them.^2^^,^^3^ Thus the RPOA introduces a new notion of intervention (one not yet properly defined, as noted above), and thus a new term is appropriate.

Third, Vanderweele *et al.* accuse us of a logical fallacy, although they do not show that we have committed the fallacy they specify. We do not agree that we have committed this fallacy. Their substantive point appears to be that what we call the RPOA is not, in fact, a theory of causation or causal inference *in toto*, but is rather the study of a certain subset of cases of causality: those counterfactuals that correspond to well-defined interventions. This also amounts to a ‘straw man’ charge.

In response, we distinguish three possible positions that might be taken by VanderWeele *et al.*:

- variables such as obesity status and race (along with many other ‘states’) cannot be considered causes in their own right, in the absence of well-defined interventions upon them;
- such variables may be causes, but at this stage they are not being incorporated into causal inference theory, which is focusing on the more tractable problem of estimation of causal effects of variables upon which there are interventions which are well-specified;
- they are causes, but cannot be incorporated into causal inference theory as it is currently being developed, although they can be incorporated into broader approaches (perhaps via a pragmatic pluralism).

Contrary to what they contend in their letter, RPOA advocates (at least sometimes) appear to take position (i). For example: ‘Causal effects cannot be defined, much less computed, in the absence of well-defined interventions’.^2^ We believe that this is either an expression of (i) or else reasonably interpreted as such. Instead of asking ‘How much cardiovascular disease is caused by obesity?’ we are told that we should ask ‘How much cardiovascular disease can be prevented by a specific intervention which reduces obesity?’ If this is a directive, and not merely a choice, then (i) appears to be the position. The restriction of (i) may be reasonable enough in devising policy,^7^ but it would be a remarkable extension to apply it to assessing causality itself.

In contrast, in their letter VanderWeele *et al.* seem to take position (ii) and argue that they are simply focusing on the estimation of causal effects of well-defined interventions (or their observational equivalents).

Our position is (iii). However, (ii) and (iii) are close. If Vanderweele *et al.* are happy to endorse (ii), and concede that their methods involve just one approach to generating information relevant to causal inference for a particular type of cause, then we applaud their efforts, and agree that they have made major methodological advances in this area.

Nonetheless, our concern remains that in practice the methods in question are being proposed and understood as a general theory of causal inference, which we call the RPOA. We maintain, and in their letter Vanderweele *et al.* acknowledge, that as a general theory the RPOA leaves out or mishandles many of the key variables that epidemiologists wish to study. When training epidemiologists to study causes, their excellent methodological work on estimating the effects of interventions (real or hypothetical) needs to be studied together with other approaches to causal inference, as outlined in our paper. Books bearing titles such as *Causal Inference* might more accurately bear titles such as *Estimation of Causal Effects of Variables Under Interventions.*

We thank Chiolero for his letter,^8^ and draw his attention to each of our independently published works criticizing aspects of risk factor epidemiology for vagueness.^9–13^ We share some of the concerns raised in the letter, and we agree that addressing concerns like these may be part of the goal of the RPOA; but we do not accept that dissatisfaction with aspects of risk factor epidemiology warrants restricting attention to a subset of causal questions.

We thank Schooling *et al.* for their letter,^14^ and we appreciate their remarks and sentiments. We agree that the methodological revolution in epidemiology in recent years holds out hope and promise for the discipline. We wish to reiterate that we are strong supporters of the development and use of the methods in question; our only concern is that they are used correctly and, to this end, that they are correctly understood.
